# Efficacy of an incentive intervention on secondary prophylaxis for young people with rheumatic fever: a multiple baseline study

**DOI:** 10.1186/s12889-019-6695-3

**Published:** 2019-04-05

**Authors:** John G. Oetzel, Chunhuan Lao, Michelle Morley, Kathy Penman, Maree Child, Nina Scott, Miina Karalus

**Affiliations:** 1 Waikato Management School, University of Waikato, Private Bag 3105, Hamilton, 3240 New Zealand; 2 Pinnacle Midlands Health Network, Norris Ward McKinnon House, 711 Victoria Street, Hamilton, 3240 New Zealand; 3 Waikato District Health Board, Pembroke Street, Private Bag 3200, Hamilton, 3240 New Zealand

**Keywords:** Rheumatic fever, Māori, Incentives, Medication adherence, Young people

## Abstract

**Background:**

Acute rheumatic fever in New Zealand persists and is a barometer of equity as its burden almost exclusively falls on Māori and Pacific Island populations. The primary objective of this study is to determine whether an incentive programme will result in increased secondary prophylaxis injections over a one-year period compared to a baseline period prior to the intervention.

**Methods:**

The evaluation used a multiple baseline study to determine whether an incentive consisting of a mobile phone and monthly “top-up” (for data/calls) resulted in increased injections, increased texts/calls with nurses, reduced number of visits to get a successful injection, less medicine wasted, and increased nurse satisfaction. Participants were 77 young people (aged 14–21) on an acute rheumatic fever registry in Waikato region, New Zealand classified as either fully adherent (all injections received and no more than one late) or partially adherent based on injections at baseline.

**Results:**

There was a sharp increase in injections for intermittent patients post-intervention and then a slight decrease overtime, while fully adherent patients maintained their high rate of injections (*p* = .003). A similar pattern for nurse satisfaction emerged (*p* = .001). The number of calls/texts increased for all patients (p = .003). The number of visits went down for partially adherent patients and up for fully adherent patients (*p* = .012). The overall incremental cost-effectiveness was $989 per extra successful injection although costs increased sharply toward the end of the intervention.

**Conclusions:**

Incentivising secondary prophylaxis appears to have a strong impact for partially adherent patients, particularly during the early periods following the initiation of the intervention. Enhancing communication with patients who returned to care may result in more sustainable adherence.

**Trial registration:**

Retrospectively registered: Australia New Zealand Clinical Trials Registry ACTRN12618001150235, 12 July 2018.

## Background

While acute rheumatic fever (RF) has declined to near zero in most developed countries, it persists in New Zealand (NZ) with 168 new hospitalizations in 2018 (3.6/100,000) [1]. RF is a barometer of equity and its burden almost exclusively falls on Māori and Pacific Island populations [2–5]. One study found that more than 90% of diagnosed cases are in Māori and PI peoples with a 30–40 times greater likelihood of being diagnosed with RF than the European/other population [5]. RF is associated with poverty and poor housing, including inadequate heating, poor insulation and crowded living conditions [1, 3].

RF is an autoimmune reaction to untreated group A streptococcal pharyngitis. RF results in swelling and inflammation of the heart, skin, brain and joints. Rheumatic heart disease (RHD) results from the inflammation of a single episode or recurrent RF; it is damage to one or more heart valves from stretching or scarring so the valves do not work properly. This may result in a need for value replacement surgery [1]. RHD has significant impact on patients as it may result in premature death in adults and is the primary cause of morbidity and mortality for people who have had RF [6].

In New Zealand, individuals with an episode of RF receive secondary prophylaxis (SP); specifically an every 28-day injection of antibiotics (i.e., benzathine penicillin G or ‘bicillin’) for a minimum of 10 years or until age 21 (whichever is longer) for those with no/mild rheumatic heart disease [1]. SP prevents up to 97% of recurrence [7, 8] and has also been shown to be the most cost effective strategy to prevent RHD [9].

Non-adherence to the injection schedule is a challenge placing individuals at high risk of RF recurrence and/or RHD [10–12]. Evidence suggests 77% of recurrence occurs within the first 7 years post-initial episode of RF [13]. The cost of non-adherence is large–60-70% of those with RF who do not have monthly penicillin injections will go on to develop permanent heart valve damage/RHD requiring costly surgery and increased health burden [9].

Researchers have noted a number of factors associated with SP adherence in developing countries and underserved populations in developed nations [11, 12, 14–19]. In New Zealand, Barker and colleagues [20] identified three levels of enablers and barriers to SP adherence through an in-depth interview study of young people (aged 14–21) with RF. First, access and resources include district nurses (DNs) coming to patient’s work or home to administer injections as an enabler. In contrast, lack of income and getting time off work were barriers. In particular, lack of income made access to mobile phones, and hence getting in contact with DNs, a challenge. Second, at the relational level, support from family and friends was an enabler, while a lack of support was a barrier. Third, at an individual level, high understanding of RF and high personal responsibility were enablers of SP adherence. In contrast, fear of and pain experienced with injections were barriers.

Addressing this range of barriers for SP adherence can be challenging for clinicians and researchers. Monetary incentives (including vouchers or products) for addressing undesirable health care behaviours, including medication adherence in adolescent populations, has gained popularity [21–23]. Monetary incentives and other rewards-based programmes have a high level of acceptability for adolescents [24]. More importantly, there is evidence that incentives produce positive health outcomes for young people on such issues as substance abuse [25], physical activity [26] and fruit and vegetable consumption [27]. However, incentives are not without challenges and some argue that they are paternalistic, coercive, and undermine agency [28]. Further, incentives may “crowd out” other motivators for health and other behaviour such as altruism, social norms and individual motivation [29, 30].

Monetary incentives are currently not used in clinical settings in New Zealand; we also could not locate any literature demonstrating it use in other locations. Thus, the primary objective of the project is to determine whether an incentive programme will result in increased SP injections for young people on an RF registry over a one-year period compared to a baseline period prior to the intervention. In addition, the analysis will calculate the incremental cost-effectiveness ratio for delivering the intervention. Secondary aims were to determine whether the following desired effects were achieved post-intervention relative to pre-intervention period: a) increased texts or phone calls between DNs and patients; b) reduced number of visits to deliver regular injections; c) reduced waste of medicine; and d) increased DN satisfaction. The increase texts/calls is desirable because it can reduce the number of no show appointments and save nurses time (i.e., reduced visits to deliver injections). The reduced wasted medicine is important in the context of a world-wide shortage of bicillin from 2014 to 2016 [31]. DN satisfaction can have implications for quality of relationships with patients which some studies have shown to be important for adherence [17, 32].

The region where this study took place is the Waikato District Health Board. The Waikato is the central region of the North Island of New Zealand and includes a larger proportion of Māori and small portion of PI relative to the whole of New Zealand. It is predominantly a rural region with one moderate-sized city (Hamilton) where the primary hospital is located. Rural regions are staffed by DNs who cover large areas with small populations in cities and towns and hence spend a lot of time in transportation. The patients in the RF registry generally have low socio-economic backgrounds and a portion (about 20–25%) of these patients are highly mobile and difficult to track and keep in touch with, particularly when they do not have mobile phones.

## Methods

### Research design

The research design was a multiple baseline following participants for a period of 15 months (3 months prior to intervention and 12 months after). In addition, the project had participatory elements with a steering group formed and composed of population health practitioners, district nurses, Māori health experts and young people with RF. The steering group collaborated on the development and implementation of the intervention. The project was retrospectively registered in the Australia New Zealand Clinical Trials Registry (ACTRN12618001150235).

### Participants

Patients (*N* = 85) between 14 and 21 years on the Waikato RF Registry receiving SP were eligible for the intervention. On the total 85 eligible, 77 agreed to participate; 17 of these participants withdrew at various stages due to being discharged (i.e., completing their injections, *n* = 2) or moving to a new location where progress could not be tracked (*n* = 15).

The three-month period prior to the start of the intervention was used to determine patient adherence status. Patients who received all three injections and were late on no more than one of the injections were classified as fully adherent (*n* = 39). Patients who missed one or more injections or were late on two or three injections were classified as partially adherent (*n* = 38). We considered using the existing registry for categorising patients’ adherence status, but inconsistencies in data entry directed us to use the baseline period. Specifically, data entry was not up-to-date or did not reconcile with supervising nurses’ knowledge about certain patients.

Given the imbalance in baseline (3 months) and post-intervention (12 months), we broke the post-intervention into four quarters to have comparable time periods. The following are the quarterly participation rates: a) Pre: Fully adherent (39); Partially adherent (38); b) Post 1st Quarter: Fully adherent (38); Partially adherent (36); c) Post 2nd Quarter: Fully adherent (37); Partially adherent (36); d) Post 3rd Quarter: Fully adherent (37); Partially adherent (34); and e) Post 4th Quarter: Fully adherent (34); Partially adherent (26). Key demographic information for participants included the following: average age of 17.7 (SD = 2.00), 47% female, 82% Māori (including 3 Cook Island Māori), 15% Pacific, and 3% other. The average length of time on the registry was 43.64 months (SD = 30.74, median = 48). There was no difference in the length of time on the registry for adherence status.

At the end of the intervention, 10 patients (fully adherent = 4; partially adherent = 6) completed an in-depth interview to reflect on the intervention (these patients also completed an interview at the start of the intervention although it was a convenience sample). In addition, seven district nurses (DN) who administered the intervention and collected monthly data also completed an in-depth interview about their experience. The interviews concentrated on the participants’ perspective of the overall programme and concentrated on how communication was impacted and the implementation of the programme.

### Intervention and procedures

The intervention was a new mobile phone at the beginning of the intervention period and a top-up card ($20 for texts, calls, and data) when the patients received their injection. Patients on contracted plans were offered an alternative incentive of $20 in grocery vouchers (*n* = 4). The phone and top-up were selected as the incentive because a) it was an appealing incentive; b) it addresses a social economic need for the high proportion of young people with income challenges; and c) DNs identify getting in contact with patients as a key challenge.

Participants were recruited to the study by the DNs through text, phone and word-of-mouth. Patients who could not be reached by the DN were recruited by a member of the research team. The research procedures received ethical approval by the Northern A Health and Disability Ethics Committee (16NTA33) and the Waikato DHB Māori Research Review Committee. The baseline period was conducted for three months prior to consent. Once patients and/or parents provided written consent (if participants < 16 years of age), the patients then received their phone and then received the $20 top-ups after their regularly scheduled injection and data was included for research. Multiple attempts were made to contact patients after a missed injection and the top-up was provided as soon as the patients received their injections (even when late). The intent of the programme was for the phone to lock out after 28 days from the last top up (and injection), but unfortunately this function was unable to be delivered upon by the phone provider. As such the incentive in relation to timeliness potentially lost the intended impact.

### Measures

The primary outcome measure was the number of injections received in a three-month period. This was averaged to three even though injections are received every 28 days in order to have a standard comparison across the quarters. The quarter was chosen for analysis as it was the length of the baseline period. Secondary outcome measures included number of in-person visits by the DN prior to successful injection, number of texts or phone calls placed by the DN to achieve the injection, number of wasted injections (from taking medicine to an appointment that was not kept by a patient), and DN satisfaction with the injection process. The DN satisfaction was measured on a single item ranging from 1 completely dissatisfied to 10 completely satisfied. All measures were recorded by the DN for each injection period (including date injection received and scheduled) and given to a member of the research team who entered the data. Data entry was checked by a second team member and missing data was followed up by this team member. Patients were not asked to provide any information nor complete any forms during the regularly scheduled visits.

### Data analysis

Three types of data analysis were undertaken to address the specific aims. For the first and second analyses, the three-month period before the intervention was used as a baseline, and the intervention arm was the three-month increments after the intervention. First, to address the outcomes, a repeated measures analysis of variance was completed. The dependent variables were the primary and secondary outcomes with time (pre-intervention quarter, and the four quarters post interventions) and injection status (fully adherent vs. partially adherent) as the independent variables. All analysis was completed with SPSS 25.0 (IBM, New York). Missing data for two patients rendered the final analysis to 58 participants for the outcomes including all time periods. We compared patients with complete data for all four time periods and those who withdrew in the last quarter (i.e., either transferred or completed injections after three quarters) and found minimal differences; hence we used the entire time period of the study. Second, to address cost-effectiveness, a decision tree was built for the incentives in increasing the adherence of injections amongst Waikato young people with RF. The cost-effectiveness analysis was from the perspective of the public provider. Only direct costs to providers were included, and costs to patients were not considered. The direct costs to providers included costs of visit before injection, the medicine, materials for injection, transport, nurses’ time, phones, top-ups and phone calls from the nurses. The unit costs and data sources of these resources are outlined in Table 1. Incremental analysis was performed in terms of incremental cost-effectiveness ratio (ICER) by dividing the incremental costs with the incremental injections by applying the intervention [33, 34]. The main outcome was cost per extra successful injection. One-way sensitivity analysis was performed to test the robustness of the results, by increasing or decreasing 20% of the unit costs or amount of medical resources. Third, for the qualitative analysis to address the participant and DN perspectives of the interventions, framework analysis was employed [35].

**Table 1:** Unit costs and data sources

Cost elements		Unit cost	Data source
Visit before injection	Transport and nurse’s time	$103.50 per trip	Waikato DHB
Successful visit for injection	Transport and nurse’s time	$103.50 per trip	Waikato DHB
Cost per Penicillin injection	Including injection materials	$50 per injection	Waikato DHB, Online Pharmaceutical Schedule
Cost per Penicillin injection wasted	Not including injection materials	$31.5 per injection	PHARMAC Online Pharmaceutical Schedule
Phone		$399 per phone	Waikato DHB
Top up or grocery voucher		$20 after one injection	Waikato DHB
Phone call from the nurse	Nurse’s time for making phone calls	$5 for a 5 mins phone call or text	MECA salaries for community DNs

Note: overhead cost was included

## Results

### Intervention outcomes

#### Injections

**Fig. 1:**
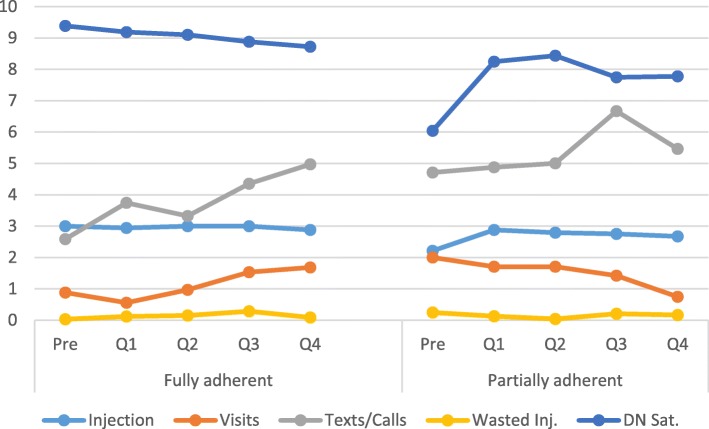
Plots of means for outcome variables comparing fully adherent to partially adherent patients over five time periods. Note: Y axis refers to number of injections, visits, texts/calls and wasted injection; DN satisfaction is rated on a scale from 0 to 10

Table 2 displays the means and standard deviations for each of the outcome variables for the fully adherent and partially adherent groups for each of the five time periods. For injections, there was a significant baseline between-subjects effect [F(1,56) = 15.42, *p* < .001] and a time X baseline status within-subjects effect [F(4,224) = 4.14, *p* = .003]. The between-subject effect illustrates that those rated as fully adherent had a higher number of injections than those in the partially adherent status. The within-subject effect demonstrates that fully adherent patients maintained their high rate of injections, while partially adherent patients had a sharp increase after the intervention then slightly decreased over time (i.e., a quadratic effect, [F(1,56) = 6.32, *p* = .015]). See Fig. 1 for a display of the within-subjects effect.

**Table 2:** Means and Standard Deviation of Outcome Variables

	Time Period	Injections		Visits before Successful Injection		Texts/Calls		Wasted Injections		DN Satisfaction	
		*M*	*SD*	*M*	*SD*	*M*	*SD*	*M*	*SD*	*M*	*SD*
Fully adherent	Pre	3.00	0.00	0.88	1.39	2.59	2.11	0.03	0.17	9.39	1.07
	Q1	2.94	0.24	0.56	0.82	3.74	3.58	0.12	0.33	9.19	1.03
	Q2	3.00	0.00	0.97	1.36	3.32	1.36	0.15	0.70	9.10	1.37
	Q3	3.00	0.00	1.53	3.28	4.35	2.73	0.29	0.80	8.88	1.50
	Q4	2.88	0.41	1.68	2.42	4.97	3.87	0.09	0.29	8.72	2.06
Partially adherent	Pre	2.21	1.14	2.00	3.44	4.71	6.89	0.25	0.90	6.04	3.78
	Q1	2.88	0.61	1.71	2.73	4.88	2.52	0.13	0.34	8.25	2.31
	Q2	2.79	0.59	1.71	2.97	5.00	4.65	0.04	0.20	8.44	2.20
	Q3	2.75	0.74	1.42	2.24	6.67	4.51	0.21	0.51	7.75	2.72
	Q4	2.67	0.87	0.75	1.57	5.46	4.65	0.17	0.56	7.78	2.84

#### Wasted injections

There were not any significant between-subjects or within-subjects effects for wasted injections. Overall, there was a 4.9% rate of wasted injections at baseline and a combined rate of 4.4% post-intervention (with 3.2% the lowest in Quarter 1). There were no significant differences between baseline and any post-intervention period.

#### Visits

There was a significant baseline between-subjects effect [F(1,56) = 5194.15, *p* < .001] and a time X baseline status within-subjects effect [F(4,224) = 3.23, *p* = .013]. The between-subject effect shows that those rated as fully adherent had fewer visits with the DN than the partially adherent group. However, the within-subject effect qualifies this effect and reveals that the number of patient visits with fully adherent patients increased over time post-intervention, while the number of visits with partially adherent patients decreased over time such that by the end, fully adherent patients had more visits than partially adherent patients (i.e., a linear effect, [F(1,56) = 6.79, *p* = .012]; see Fig. 1).

#### Texts/calls

There was a significant baseline between-subjects effect [F(1,56) = 6.11, *p* = .016] and a time within-subjects effect [F(4,224) = 2.98, *p* = .020]. The between-subjects effect illustrates that more texts/calls were made to partially adherent patients than fully adherent patients. The within-subject effect demonstrates that the number of texts/calls increased from baseline throughout the study duration (i.e., a linear effect, [F(1,56) = 9.65, *p* = .003]).

#### DN satisfaction

There was a significant baseline between-subjects effect [F(1,56) = 16.71, *p* < .001] and a time X baseline status within-subjects effect [F(4,224) = 4.74, *p* = .001]. The between-subject effect illustrates that those rated that DN satisfaction was higher with fully adherent patients than with those in the partially adherent status. The within-subject effect shows DN satisfaction for fully adherent patients slightly decreased over time, while it sharply increased for partially adherent patients post-intervention and then slightly decreased over time (i.e., a quadratic effect, [F(1,56) = 5.65, *p* = .021]; see Fig. 1).

### Incremental cost-effectiveness ratio

The intervention increased the number of successful injections and total costs (Table 3). The ICER was relatively small for the 1st quarter and the 2nd quarter after intervention ($741 and $723 per extra successful injection), but almost doubled in the 3rd quarter ($1322 per extra successful injection) and tripled in the 4th quarter ($2219 per extra successful injection). The overall ICER for the 12-month period was $989 per extra successful injection.

**Table 3:** Medical resources and cost-effectiveness results between the pre-intervention and post-intervention

Results		Q1	Q2	Q3	Q4	Overall
Number of eligible patients		74	73	71	60	77
Number of phones allocated^†^	Post-intervention only	82	82	80	72	86
Number of visits before successful injection	pre-intervention	107	109	106	81	403
	Post-intervention	86	96	105	75	362
Number of phone calls/texts before successful injection	pre-intervention	285	286	273	209	1053
	Post-intervention	369	362	426	302	1459
Number of injections wasted	pre-intervention	10	10	9	7	36
	Post-intervention	7	8	15	7	37
Number of successful Injections	pre-intervention	194	192	190	160	736
	Post-intervention	212	212	201	165	790
Total costs	pre-intervention	$42,594	$42,499	$41,785	$34,209	$161,086
	Post-intervention	$55,928	$56,960	$56,324	$45,303	$214,514
Incremental Effectiveness (extra successful injections)		18	20	11	5	54
Incremental Cost		$13,335	$14,461	$14,539	$11,094	$53,428
ICER (cost per extra successful injection)		$741	$723	$1322	$2219	$989

^†^ Patients were given a second phone if the first one was lost or broken; Note: Total costs include phone costs even though they were donated to the project

The sensitivity analyses (Table 4) show that the number of visits before successful injections post-intervention has the highest impact on the overall ICER. Increasing or decreasing 20% of the number of visits before successful injections results in 14% increase or reduction in the costs per extra successful injection. In contrast, the number of phone calls and texts before injection or the unit cost of phone call or text has a small impact on the ICER. Increasing or decreasing 20% of the number of phone calls and texts increases or decreases only 2.7% of the costs per extra successful injection.

**Table 4:** Results of one-way sensitivity analyses

Factors		Incremental costs	Overall ICER	Difference compared to the baseline ICER
Amount of medical resources post-intervention				
Number of phones allocated	+ 20%	$59,732	$1106	11.8%
	-20%	$47,124	$873	−11.8%
Number of visits before injections	+ 20%	$60,921	$1128	14.0%
	−20%	$45,935	$851	−14.0%
Number of calls or texts before injection	+ 20%	$54,887	$1016	2.7%
	−20%	$51,969	$962	−2.7%
Number of injections wasted	+ 20%	$53,661	$994	0.4%
	−20%	$53,195	$985	−0.4%
Unit costs of medical resources pre/post-intervention				
Unit cost of phone	+ 20%	$59,732	$1106	11.8%
	−20%	$47,124	$873	−11.8%
Unit cost of visit for injection	+ 20%	$53,697	$994	0.5%
	−20%	$53,159	$984	−0.5%
Unit cost of Penicillin (not including injection materials)	+ 20%	$53,974	$1000	1.0%
	−20%	$52,882	$979	−1.0%
Unit cost of top up/ grocery voucher	+ 20%	$56,588	$1048	5.9%
	−20%	$50,268	$931	−5.9%
Unit cost of 5 min phone call or text	+ 20%	$53,834	$997	0.8%
	−20%	$53,022	$982	−0.8%

### Post-intervention interviews

Two themes were identified in the post-intervention interviews: communication and implementation. Direct quotes are used to illustrate these themes and included names are pseudonyms. In regards to communication, patients viewed the intervention as an enabler of effective communication with DNs. Patients noted that the phone and top-up provided a resource they did not have before:

It was helpful for the nurses to get a hold of me and things … they did because I didn’t own a phone and they always had to ring around to like all my family member to see where I was. (Manaia, partially adherent).

Yea yea, that’s the main thing; cause a lot of the times I couldn’t text back like when they are there. And they didn’t really call me much, so it was hard to get a hold of them because I didn’t have credit… since they gave me the top up I couldn’t not text back. I didn’t have a reason not to text back. (Hohepa, partially adherent).

About half of the nurses also agreed that it facilitated good interaction with patients. For example, Memory (DN) explained,

I was able to get a hold of the people because they had a phone. Every month they could receive the texts. I could arrange times to visit, and I caught them easier because some of them don’t have the money to have a phone. So they were hard to keep contact with because with the phone numbers changed all the time or they lost the phone or it was there brother’s phone or their father’s phone or something.

The other half of the nurses thought that communication did not change. Tracey offered, “Yeah, in my experience it made no real difference...They were difficult; some patients were difficult to contact prior to the project. And those same patients are still difficult to contact during and after the project.” Stephanie stated, “Put it this way, it didn’t change the issues that we had with contacting the patients even though they had a phone and top-ups. We still had to chase them.”

The second theme focused on implementation factors that served as challenges to the programme’s effectiveness. Patients described that oftentimes top-ups were not available or they received a duplicate code that would not work again:

Three times maybe that they couldn’t find it or something, but I got in touch with the main doctor and she ended up sending it through to me. Um yea it’s just that our hospital is just unorganised (Maddison, fully adherent).

I think the last couple of weeks I never got my top-ups…And I like, I keep telling them that. The nurses never, they keep saying that they’ll give it, but they’re only allowed to give one (Abel, partially adherent).

Maybe just make sure you have the top-ups every time, because I haven’t got it in the last 5 months…Oh they weren’t too bad with like communicating, just with the top-ups. They just were not good at all. It’s like they just really didn’t care*.* (Hohepa, partially adherent).

These factors were not perceived as inhibitors to participation or receiving the injection; rather they were described as a hassle and the focus of the ire was toward the administrators of the programme not the nurses. Further, our data suggests that it occurred in less than 10% of the cases and a top-up was always provided.

The key implementation factor for many nurses was that they disagreed with providing the incentive even if patients received injections late. They felt it rewarded negative behaviour.

I’m not the only one but it was like giving a reward for negative behaviour. I know it was supposed to encourage the opposite behaviour…I didn’t think it was a good idea. Well the negative behavior was they wouldn’t come for their injections on time. So if they came on time we would give them this top-up ok? But even if they were still overdue, and didn’t come on time, and they’re how many days overdue, and we still gave them the top-up. (Stacey).

Yeah, I thought it was a negative that we were essentially rewarding…there was no consequence for them. They still received their top-up whether they received their injection on time or not. So there was no real incentive for them to actively get in touch with us or be proactive about having their injection on time because they got it anyway; so they didn’t care. So they could still have it, we could still be chasing them for two weeks after the due date and they would still get their top-up. So there was no, there was no incentive on their part to be more compliant. (Tracey).

Nurses felt that the incentive would have been more effective if they could withhold top-ups if late injections were administered. As noted in the methods, the intention was to have the phone be locked if they did not receive their injection, but this feature did not work.

## Discussion

The primary objective of this study was to increase the number of SP injections for patients on a RF registry and determine the ICER. Secondary outcomes were to increase the number of texts/calls and reduce the number of visits between patients and DN; further desired was a reduction in wasted injections and increase in DN satisfaction. The findings generally supported the objectives of the study although there were distinct differences for patients who were identified as fully adherent versus partially adherent at baseline.

The incentives had an impact on injections, but not on the number of wasted injections. The intervention had an impact overall (*p* < .01) although this was qualified by a significant time X baseline status interaction effect. Specifically, the number of injections for fully adherent patients were by definition high and generally maintained that level over the course of the project. The number of injections for partially adherent patients was moderate initially and had a sharp increase post-intervention with levels slowly tapering off over time, but still above the baseline levels. These results demonstrate that incentives have a significant increase in desired behaviour and consistent with prior research in other contexts [21, 23, 26, 27]. The lack of impact on wasted injections is likely because DNs did not take medication to visits where they were not reasonably sure that a patient would show up and hence there were minimal situations to waste an injection. This may be due to the worldwide shortage of bicillin that impacted New Zealand at the beginning of the study [31].

The overall ICER for the 12-month period was $989 per extra successful injection, but this average was inflated by the costs in the second half of the intervention period. Costs in the 1st and 2nd quarters were below $800/incremental injection. The main reason for these increased costs is the number of patients who transferred and thus there were not incremental increases in injections over the baseline period. Further, to reduce the costs per extra successful injection, we need to reduce the number of visits before successful injections, and have more phone calls or texts to contact the patients to ensure successful visits for injections. If the number of visits before a successful injection is reduced by 50% and the number of phone calls or texts increases by 50%, the overall ICER can be reduced by 28.2% (to $710 per extra successful injection). It is important to note that we included the cost of the phone in these calculations even though they were donated by the phone provider. Removing the phone costs results in an ICER of $354 per extra successful injection.

These data do not allow us to make a concrete conclusion about whether the intervention was cost-effective because of the limited time frame although some context can help with speculation and guiding future research. Often with cost-effectiveness analysis, there is desire to identify whether it impacts quality-adjusted life year [34, 36]. This would require following people for a significant amount of time to determine whether significant adverse event occurred (e.g., recurrent RF, valve surgery, death). A study of the mortality and cost of RF and RHD in New Zealand found that 71% of the annual $12 million (2009–2010 NZ dollars) in costs occurred for individuals 30 or more years of age [6]. Further, 28% of the total costs were for hospital admissions and 72% were for heart valve surgery. If the intervention prevented a later valve surgery, the current study’s ICER may be well worth it given these costs. Additionally, there were an average of 159 deaths each year with Māori and Pacific peoples having a 5–10 times greater mortality rate [6]. Thus, there are health equity issues to be considered in making these estimates. Unfortunately, we did not have sufficient funding, nor is there a current infrastructure (i.e., nurses are not required to make monthly entries to the registry), to follow patients for a longer time period even to determine even if some contracted RHD which is the key aspect of the progression from RF to heart valve surgery [13, 15]. Modelling this information from existing data could be done and is beyond the scope of the current study. Future research can be guided by these baseline costs and ways to reduce the costs.

Positive results were found in the number of texts/calls and visits. For all patients, the number of texts and calls increased post-intervention as the phone enabled inexpensive contact between patients and DNs. This was corroborated by patient interviews and some of the DN interviews. The results also illustrate that the number of visits prior to injection decreased for partially adherent patients, but actually increased for fully adherent patients. Thus, the intervention had an unintended consequence, and given the sensitivity analysis, this consequence has detrimental impacts to costs although not for outcomes. We speculate that the reason for the increased visits was related to the administration of the incentives although we cannot state with certainty.

Finally, the intervention had a positive impact on DN satisfaction working with partially adherent patients. The relationship of DN satisfaction mirrored that of the number of injections; specifically, satisfaction with fully adherent patients was high and had a slight decline over time, while for partially adherent patients satisfaction was moderate and sharply increased post-intervention and then had a slight decline, but remained above baseline. Presumably, the positive impact on SP also resulted in positive DN satisfaction as it made patients easier to contact and hence reduced the job demands for the nurses [37, 38]. However, there was slightly declining DN satisfaction for fully adherent patients. The fully adherent patients were initially high and DNs did not have administrative responsibilities with them (i.e., data collection and top ups). Further, the interviews with patients also noted some challenges with administration related to the top-ups (although not a widespread problem). Hence, the DNs had more work for no increase in patient outcomes. Thus, these factors may also have resulted in declining DN satisfaction for fully adherent patients. These findings may have impacts on the patient-nurse relationships and that potential could have negative impacts on adherence given that some prior research has found this relationship to be an important enabler of SP [17, 32]. However, our own research with the current patient population that the relationship with the nurse is not the key enabler, but rather family support is most critical so future research is warranted [20].

The results of this study have some important practical implications for SP of RF in New Zealand. The intervention had positive impacts for partially adherent patients and actually brought some patients back to care (based on interview responses and DN notes). Conservatively, the DNs suggested (based on clinical notes) that at least 16 patients were re-engaged to care when they were thought to be non-adherent (and not just partially adherent). Such re-engagement is very important given that small doses of bicillin can be important at preventing recurrent RF [7, 8, 15]. It increased access to inexpensive communication and this is important as this patient base has lower socio-economic status and often do not have credit or data to contact their DN or reply to texts as noted in the interviews with patients. Thus, the intervention is potentially equity enhancing given the inequitable distribution of income for this patient population [39]. This is important in the context of potential valvar surgery for patients who advance to rheumatic heart disease due to lack of adherence to SP.

Unfortunately, the intervention only had minimal positive impacts for fully adherent patients. Specifically, it increased texts and calls with the DN, but it also resulted in more visits. Thus, this appears to be additional costs for this patient population and perhaps only non-adherent patients should be incentivised. This incentive certainly raises an ethical discussion about whether it is appropriate to only incentivise patients who are not adherent and yet the results suggest the positive outcomes are only for non-adherent patients. These ethical discussions need to be considered into the impact of incentives “crowding out” internal motivations for behaviour [29, 30]. In fact, some research demonstrates this crowding out exacerbates after the incentive is removed [29]; this effect was beyond the study design as we did not collect data after the incentive was stopped and can be addressed with further research.

Beyond the simple incentive, there are key elements of the administration that should be considered. First, the DNs who participated in interviews suggest that providing top-ups to patients who were late at the very least rewarded, and possibly even encouraged, negative behaviour. More importantly, DN engagement with patients who return to care needs to be given careful consideration. Unfortunately, 71% of the patients who transferred or did not complete the study were from the partially adherent group. In other words, the incentive brought them back to care, but only maintained adherence for 6–9 months for some of them. This is a key opportunity for DNs to (re)build a relationship of trust with the patients to encourage long-term adherence to SP. This recommendation is consistent with research demonstrating that enhanced communication and incentives provide a strong impact on health outcomes [25]. However, such engagement also needs to be considered in the context of a large workload for DNs. It also may suggest the need for a larger scale study across District Health Boards in New Zealand since this partially adherent group is highly mobile and a linked approach to SP may prove more effective.

This study is not without limitations. First, other than patient interviews, we did not collect any data to explore patients reasons for entering the programme or what happened when they left. Thus, explanation of why the programme worked is limited. Second, the study had a fairly high (22%) attrition rate by the final quarter. This attrition does raise concern about the sustainability of the impacts of the incentives and hence the need to couple the intervention with a strong relationship with the DNs. Third, we did not have a comparison/control group and thus we cannot know for certain whether the impacts are directly due to the intervention. Finally, resources limited the length of the baseline period and any post-intervention data collection. A longer baseline period would better establish the predominant behaviour of patients, while a post-incentive data collection would help to determine if behaviour is maintained. Such data would help make a stronger conclusion about the cost-effectiveness of the intervention.

## Conclusions

This study sought to determine if an incentive of a mobile phone and monthly top-ups would increase SP injections for patients on a RF registry. This incentive demonstrated positive impacts for patients determined to be partially adherent. It provided patients with a resource that facilitated effectively and timely communication with nurses. It can have positive short-term impacts and potential long-terms impacts with effective engagement by clinical staff. There is a need to effectively administer the programme to not inadvertently impact nurse-patient relationships. Ethical considerations and future research are needed to determine whether incentives should be expanded to all RF patients, particularly those that are fully adherent with their injections, and whether late injections should be fully, partially or not incentivised.

## Abbreviations

DN: District Nurse
ICER: Incremental Cost-Effectiveness Ratio
NZ: New Zealand
RF: Acute Rheumatic Fever
RHD: Rheumatic Heart Disease
SP: Secondary Prophylaxis
